# Brain frailty associated with stroke events in anterior circulation large artery occlusion

**DOI:** 10.1186/s12883-024-03566-7

**Published:** 2024-03-18

**Authors:** Jing Tian, Kun Zhang, Junzhao Cui, Jin Qin, Binbin Wang, Lixia Zhou, Tong Li, Kailin Bu, Zhongzhong Li, Lin Liu, Qisong Wang, Si Yuan, Lina Ma, Ye Wang, Rui Wang, Chaoyue Meng, Biyi Zhou, Li Guo, Xiaoyun Liu

**Affiliations:** 1 Department of Neurology, The Second Hospital of Hebei Medical University, 215 West Heping Road, Shijiazhuang, Hebei 050000 China; 2 Department of Radiology, The Second Hospital of Hebei Medical University, Shijiazhuang, China; 3 Neuroscience Research Center, Medicine and Health Institute, Hebei Medical University, Shijiazhuang, China; 4 Department of Neurology, The First Hospital of Hebei Medical University, Shijiazhuang, China

**Keywords:** Brain frailty, Anterior circulation, Large artery occlusion, Stroke events

## Abstract

**Objective:**

To investigate the factors associated with brain frailty and the effect of brain frailty in patients with anterior circulation large artery occlusion (AC-LAO).

**Methods:**

1100 patients with AC-LVO consecutively admitted to the Second Hospital of Hebei Medical University, North China between June 2016 and April 2018 were retrospectively analyzed. The variables associated with brain frailty and stroke outcome were analyzed by ANOVA analysis, the Mann-Whitney *U* test and multiple linear regression. Based on previous research. Brain frailty score comprises 1 point each for white matter hyperintensity (WMH), old infarction lesions, and cerebral atrophy among 983 participants with baseline brain magnetic resonance imaging or computed tomography.

**Results:**

Among AC-LAO participants, baseline brain frailty score ≥ 1 was common (750/983, 76.3%). Duration of hypertension > 5 years (mean difference [MD] 0.236, 95% CI 0.077, 0.395, *p* = 0.004), multiple vessel occlusion (MD 0.339, 95% CI 0.068, 0.611, *p* = 0.014) and basal ganglia infarction (MD -0.308, 95% CI -0.456, -0.160, *p* < 0.001) were independently associated with brain frailty score. Brain frailty score was independently associated with stroke events, and higher brain frailty scores were associated with higher rates of stroke events (*p* < 0.001). However, brain frailty has no independent effect on short-term outcome of ACI in AC-LAO patients.

**Conclusions:**

In AC-LAO patients, older age, duration of hypertension > 5 years, and multiple vessel occlusion influenced the brain frailty score. Brain frailty score was independently associated with the occurrence of stroke events in AC-LAO patients.

**Supplementary Information:**

The online version contains supplementary material available at 10.1186/s12883-024-03566-7.

## Graphical Abstract

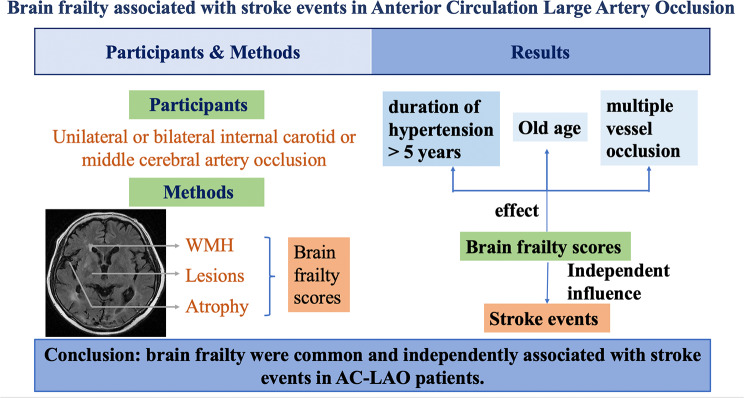

## Introduction

In patients with stroke, many clinical and imaging features are associated with long-term outcome [1–3], making it challenging to accurately predict outcome [4]. The absence of small vessel changes and presence of larger brain volumes are imaging markers of brain health [5, 6]. An increasing number of researchers believe that global brain health may also influence recovery [7], as the recovery process after stroke involves the re-optimization of cellular, structural, and vascular functions throughout the brain, while maintaining their integrity [8]. General prestroke features, including white matter hyperintensity (WMH), cerebral atrophy, and old infarct lesions, have been independently associated with poor outcome and may represent markers of brain frailty [9–12]. Brain frailty is usually conceptualized as the product of physiological changes associated with aging and the accumulation of multimorbidity of underlying brain pathology in patients presenting within the typical stroke age bracket [13]. This causes a loss of resilience to acute disease that may result from both disease and extrinsic stressors.

Anterior circulation large artery occlusion (AC-LAO) is the most common cause of ischemic stroke and that is strongly associated with prognosis [14]. It differs from the expected imaging markers of brain frailty mainly in patients with small vessel disease (SVD), some studies have suggested that patients with intracranial arterial stenosis (ICAS) may be particularly prone to coexisting SVD, which may affect outcomes in patients with ICAS [15–18]. However, there is a lack of data that compares with general patients to determine whether the factors influencing brain frailty in patients with large artery occlusion are different, and what impact brain frailty has on the prognosis of patients with large artery occlusion.

The aims of the present analysis were to assess the factors that influence brain frailty in patients with AC-LAO, and to explore the association between brain frailty and the incidence of cerebrovascular disease and outcome of ACI in AC-LAO patients.

## Methods

### Patients

Details of the study protocol, baseline characteristics, and main results have been published previously [19, 20]. In summary, we retrospectively analyzed 1100 patients with AC-LAO who were consecutively hospitalized at the Second Hospital of Hebei Medical University, North China, between June 2016 and April 2018. These patients presented with complete occlusion of at least one unilateral intracranial internal carotid artery (ICA) or middle cerebral artery (MCA) based on computed tomography angiography (CTA) or magnetic resonance angiography (MRA). Computed tomography (CT) or magnetic resonance imaging fluid-attenuated inversion recovery (MRI-FLAIR) and MRI-T2 data were obtained from 983 patients at baseline. Stroke events were defined as this hospitalization and all previous symptomatic strokes. Most patients had 1 stroke event (546, 55.5%), followed by 0 (190, 19.3%), 2 (189, 19.2%), 3 (26, 2.6%), 4 (22, 2.2%), > 5 (8, 0.8%), and 5 (2, 0.2%), of which 645 had ACI. Patients or relatives/caregivers provided informed consent, and the study protocol was approved by the Research Ethics Committee of the Second Hospital of Hebei Medical University (approval No.2018-P403).

### Brain frailty score

CT or MRI readings were performed independently by two neuroradiology specialists who assessed and documented the location and shape of any acute ischemia and the presence of pre-stroke changes, including atrophy, white matter hyperintensity (WMH), and old infarct lesions. Atrophy was scored according to a standard template, defined as 0 = absent, 1 = moderate or severe [21, 22]. We assessed the degree of brain atrophy in both the cortical and central regions; as long as any part of the brain is atrophy, the score is 1. WMH was scored separately in anterior and posterior brain regions and periventricular and deep white matter, defined as 0 = no lucency, 1 = lucency restricted to regions adjacent to the ventricles, or covering the entire region from lateral ventricle to cortex [23]. Old vascular lesions/infarcts were classified by location (e.g., cortical, striatocapsular, border zone, lacunar), and 0 = no old vascular lesions/infarcts or 1 = old vascular lesions/infarcts in any location. According to two recent studies of brain frailty [9, 10], a 3-point score was used to assess brain frailty, including 1 point for WMH, 1 point for atrophy, and 1 point for any chronic infarct or vascular lesion.

### Statistical analysis

Statistical analyses were performed with SPSS 25.0 (IBM, Corp. Armonk, NY). Continuous variables were presented as mean (SD) or median (interquartile range) and compared by 1-way ANOVA analysis or Mann-Whitney *U* test. Categorical variables were presented as percentages and compared with the χ^2^ test. Candidate risk factors for multivariable analysis were those with *p* < 0.10 in the bivariate analyses described above. Extreme values (more than 3 standard deviations) were removed before the analysis of continuous variables. Associations between baseline data and brain frailty score were assessed by multiple linear regression, as the conditions for ordinal regression were not met. The association between imaging markers of brain frailty and stroke events was analysed by ordinary multiple regression. Regression analysis was performed on certain variables based on univariate analysis, clinical experience, and previous literature results, including age, sex, medical history, baseline NIHSS score, blood lipids and homocysteine, infarct shape and location, occluded vessels, and complications. Results are reported as odds ratios (OR) or mean differences (MD) and 95% confidence intervals (CI) or standardized regression coefficients (𝛃), with significance defined as *p* ≤ 0.05.

## Results

Of the 1100 participants in the AC-LVO cohort, 983 underwent brain imaging (250 brain CT scans and 733 brain MRI scans) at the Second Hospital of Hebei Medical University. Baseline characteristics of the participants were shown in Table 1. The median age of the patients was 62 years (age range: 10–96). There were 667 men (67.9%) and 316 women (32.1%). The most common medical history was hypertension (65.4%), of which 65.0% had been present for more than 5 years, followed by history of stroke (37.1%), smoking (35.0%) and hyperlipidemia (32.0%). Among the 983 patients, the median number of stroke events was 1 (categorized as 0, 1, 2, 3, 4, 5, and > 5), and 645 (65.6%) had ACI, whose infarct locations and morphology, hospital complications, NIHSS and mRS scores at admission and discharge were recorded in detail. Among them, the NIHSS scores of 0–4, 5–20, and ≥ 21 were 263 (40.8%), 341 (52.9%), and 41 (6.4%), respectively. According to the location of the occluded vessels, patients were divided into the MCA occlusion group (53.2%), the ICA occlusion group (29.4%) and the MCA + ICA occlusion group (17.4%). Multi-vessel occlusion in the patients (*n* = 246, 25.0%) was also recorded, as well as the number of patients with concomitant anterior cerebral artery (ACA) occlusion (21.3%) and posterior circulation (PC) occlusion (20.1%). Table 1 also shows that brain frailty is associated with the most factors, including age, duration of hypertension, smoking, history of previous stroke, multiple vessel occlusion, coronary heart disease, atrial fibrillation, low-density lipoprotein cholesterol (LDL-C), apolipoprotein B (ApoB), location of occluded vessels, and PC occlusion.

**Table 1:** Demographic and clinical characteristics of study population according to brain frailty score

Variables	Total *N* = 983	Brain frailty score				***P*** Value
		0, *N* = 233	1, *N* = 338	2, *N* = 236	3, *N* = 176	
Age, y, median (IQR)	62 (14)	55 (17)	59.5 (14)	64 (12)	68.5 (11)	< 0.001
Age categories						< 0.001
≤ 55	316 (32.1)	118 (50.6)	131 (38.8)	56 (23.7)	11 (6.3)	
56∼65	334 (34.0)	69 (29.6)	124 (36.7)	93 (39.4)	48 (27.3)	
> 66	333 (33.9)	46 (19.7)	83 (24.6)	87 (36.9)	117 (66.5)	
Male, n (%)	667 (67.9)	159 (68.2)	223 (66.0)	159 (67.4)	126 (71.6)	0.634
Smoking, current, n (%)	344 (35.0)	100 (42.9)	119 (35.2)	71 (30.5)	53 (30.1)	0.016
Drinking, current, n (%)	279 (28.4)	75 (32.2)	96 (28.4)	62 (26.6)	45 (25.6)	0.439
Hypertension, n (%)	643 (65.4)	135 (57.9)	214 (63.3)	161 (68.2)	133 (75.6)	0.002
Duration of hypertension > 5y, n (%)	418 (42.5)	67 (28.8)	132 (39.1)	118 (50.0)	101 (57.4)	< 0.001
Diabetes mellitus, n (%)	251 (25.5)	42 (18.0)	95 (28.1)	60 (25.4)	54 (30.7)	0.014
Diabetes mellitus time > 5y, n (%)	110 (11.2)	15 (6.4)	43 (12.7)	29 (12.3)	23 (13.1)	0.040
coronary heart disease, n (%)	150 (15.3)	22 (9.4)	50 (14.8)	41 (17.4)	37 (21.0)	0.009
Atrial fibrillation, n (%)	55 (5.6)	5 (2.1)	15 (4.4)	22 (9.3)	13 (7.4)	0.004
Prior TIA, n (%)	67 (6.8)	17 (7.3)	25 (7.4)	12 (5.1)	13 (7.4)	0.690
Prior stroke, n (%)	365 (37.1)	7 (3.0)	123 (36.4)	132 (55.9)	103 (58.5)	< 0.001
Hyperlipidemia, n (%)	315 (32.0)	91 (39.1)	102 (30.2)	73 (30.9)	49 (27.8)	0.061
LDL-C, mmol/L, median (IQR)	2.40 (1.15)	2.67 (1.13)	2.33 (1.17)	2.26 (1.11)	2.35 (0.96)	0.004
ApoA1, g/L, median (IQR)	1.13 (0.27)	1.13 (0.31)	1.13 (0.26)	1.13 (0.26)	1.13 (0.27)	0.984
ApoB, g/L, median (IQR)	0.95 (0.31)	0.96 (0.31)	0.95 (0.33)	0.95 (0.35)	0.93 (0.31)	0.018
HCY, umol/L, median (IQR)	16.0 (9.7)	16.2 (10.6)	15.86 (9.10)	16.0 (8.4)	16.0 (9.9)	0.382
occluded vessels position						0.009
MCA occlusion	523 (53.2)	129 (55.4)	190 (56.2)	116 (49.2)	88 (50.0)	
ICA occlusion	289 (29.4)	75 (32.2)	97 (28.7)	65 (27.5)	52 (29.5)	
MCA + ICA occlusion	171 (17.4)	29 (12.4)	51 (15.1)	55 (23.3)	36 (20.5)	
multiple vessel occlusion, n (%)	246 (25.0)	40 (17.2)	80 (23.7)	71 (30.1)	55 (31.3)	0.002
ACA occlusion, n (%)	209 (21.3)	41 (17.6)	74 (21.9)	52 (22.0)	42 (23.9)	0.137
PC occlusion, n (%)	198 (20.1)	31 (13.3)	65 (19.2)	62 (26.3)	40 (22.9)	0.004
Stroke events, median (IQR)	1 (1)	1 (1)	1 (1)	1 (1)	1 (1)	< 0.001
ACI, n (%)	645 (65.6)	168 (72.1)	224 (66.3)	140 (59.3)	113 (64.2)	0.033
Frontal lobe infarction, n (%)	362 (36.8)	104 (44.6)	115 (34.0)	84 (35.6)	59 (33.5)	0.042
temporal lobe infarction, n (%)	299 (30.4)	92 (39.5)	90 (26.6)	71 (30.1)	46 (26.1)	0.005
parietal infarction, n (%)	349 (35.5)	102 (43.8)	109 (32.2)	81 (34.3)	57 (32.4)	0.024
basal ganglia infarction, n (%)	320 (32.6)	103 (44.2)	105 (31.1)	72 (30.5)	40 (22.7)	< 0.001
Posterior circulation infarction, n (%)	151 (15.4)	39 (16.8)	52 (15.4)	34 (14.4)	26 (14.8)	0.900
The shape of the lesion in the occluded vessel						0.001
No lesions or scattered	190 (19.3)	52 (22.3)	73 (21.6)	37 (15.7)	28 (15.9)	
Patchy	603 (61.3)	121 (51.9)	207 (61.2)	154 (65.3)	121 (68.8)	
Large flakes	190 (19.3)	60 (25.8)	58 (17.2)	45 (19.1)	27 (15.3)	
Complication, n (%)	167 (17.0)	36 (15.5)	44 (13.0)	47 (19.9)	40 (22.7)	0.013
pneumonia, n (%)	108 (11)	20 (8.6)	27 (8.0)	33 (14.0)	28 (15.9)	0.012
hypoalbuminemia, n (%)	28 (2.8)	5 (2.1)	5 (1.5)	9 (3.8)	9 (5.1)	0.030
NIHSS score on admission, median (IQR)	4 (7)	4 (9)	3 (7)	4 (9)	4.5 (7)	0.037
NIHSS score at discharge, median (IQR)	3 (8)	3 (8)	2 (6)	4 (8)	4 (7)	0.007
mRS score on admission, median (IQR)	2 (3)	2 (3)	2 (3)	2 (3)	2 (3)	0.062
mRS score at discharge, median (IQR)	2 (3)	2 (3)	2 (2)	2.5 (3)	2 (3)	0.004

Abbreviations: WMH = white matter hyperintensity; IQR = interquartile range; LDL-C = Low density lipoprotein cholesterol; ApoA1 = apolipoprotein A1; ApoB, = apolipoprotein B; HCY = homocysteine; MCA = middle cerebral artery; ICA = internal carotid artery; ACA = Anterior cerebral artery; PC = Posterior circulation; ACI = acute cerebral infarction

The factors in Table 1 associated with brain frailty score (*p* < 0.10) were included in multiple linear regression analysis (Table 2). Age (MD 0.371, 𝛃 0.37, *p* < 0.001), duration of hypertension (MD 0.236, 95%CI 0.077, 0.395, *p* = 0.004), and multiple vessel occlusion (MD 0.339, 95%CI 0.068, 0.611, *p* = 0.014) were independent factors. Meanwhile, univariate and multivariate analyses of factors associated with WMH, cerebral atrophy, and old lesions were performed (see appendix).

**Table 2:** Multiple linear regression of variables and brain frailty score

	n (%)	multiple linear regression	
		MD (95%CI) 𝛃	***p*** value
Age, median (IQR)^a^	62 (14)	0.371	< 0.001
multiple vessel occlusion	246 (25.0)	0.339 (0.068, 0.611)	0.014
Hypertension time (> 5y)	418 (42.5)	0.236 (0.077, 0.395)	0.004
basal ganglia infarction	320 (32.6)	-0.308 (-0.456, -0.160)	< 0.001

Abbreviations: MD = mean difference; IQR = interquartile range; ^a^ Standardized regression coefficient reported

Brain frailty score was associated with stroke events by ordinary multiple logistic regression analysis, and higher brain frailty score was associated with higher risk of stroke [brain frailty = 1 OR 2.29 (95% CI 1.58, 3.33), brain frailty = 2 OR 3.11 (95% CI 2.07, 4.68), brain frailty = 3 OR 3.81 (95% CI 2.46, 5.89)] (Table 3). In univariate analysis, brain frailty score was associated with mRS at discharge in ACI patients (*p* = 0.004), but was not independently correlated in multivariate regression analysis (see appendix).

**Table 3:** Ordinal multiple regression analysis associated with stroke events

	n (%)	logistic regression	
		OR (95%CI)	***p*** value
n	819		
Diabetes mellitus	219(26.7)	2.25 (1.29, 3.92)	0.004
HCY, median (IQR)	16 (9.70)	1.01 (1.00, 1.02)	0.047
ACA occlusion	178 (21.7)	1.48 (1.05, 2.07)	0.02
Brain frailty score			< 0.001
0	191 (23.3)	1	
1	280 (34.2)	2.29 (1.58, 3.33)	< 0.001
2	195 (23.8)	3.11 (2.07, 4.68)	< 0.001
3	153 (18.7)	3.81 (2.46, 5.89)	< 0.001

Abbreviations: OR = odds ratio; CI = confidence interval; IQR = interquartile range; HCY = homocysteine; ACA = Anterior cerebral artery

## Discussion

This study provides a large cohort to determine the influence of brain frailty on outcomes in patients with AC-LAO. Age, multiple vessel occlusion, and duration of hypertension affect the brain health in AC-LAO. Brain frailty is a risk factor for stroke events in AC-LAO.

Previous studies have shown that brain frailty is associated with outcomes after acute ischemic stroke [10], which is stronger in lacunar infarcts [9]. However, there is limited data on the influencing factors and prognostic effects of brain frailty in patients with large artery stenosis/occlusion. Hypertension is a common risk factor for atherosclerosis and is also the most common cause of cerebral small vessel disease [24]. However, this study differs from previous ones in that it includes three variables: hypertension, hypertension grade, and hypertension duration. There variables were all found to be correlated with outcome variables in univariate analysis. In multivariate analysis, only duration of hypertension > 5 years was found to be independently associated with the brain frailty. This may reflect the long-term cumulative exposure of hypertension more accurately. A 2019 study in a group aged > 60 years showed that both midlife and current hypertension were risk factors for WMH progression [25], also highlighting the effect of duration of hypertension. We also found that the severity of cerebral arteriosclerosis is associated with brain frailty, which has not been previously reported. However, arteriosclerosis is associated with white matter disease, brain atrophy and lacunar infarction [26–28].

Our results suggest that brain frailty is independently associated with stroke events, and the causes may be multiple. First, imaging changes such as WMH and old lesions are also characteristic of CSVD, are also common in patients with large artery stenosis/occlusion, and are poor prognostic factors in patients with acute ischemic stroke [29–31] and are related to cerebral perfusion [32]. Second, previous studies have shown that patients with symptomatic intracranial artery stenosis are more likely to have stroke events than asymptomatic patients, and patients with more collateral circulation have a lower risk of stroke [33, 34], which indirectly supports our findings that multiple vessel occlusion was independently associated with brain frailty, which in turn was independently associated with the occurrence of stroke events. These results suggest that brain frailty may serve as a marker of inadequate collateral compensation of the cerebral circulation in AC-LAO patients [35]. In the future, we will compare the correlation between brain frailty and the degree of collateral circulation assessed by CTA or DSA, evaluate the feasibility of using cerebral frailty to predict collateral circulation, and provide a simpler and more feasible method for the vascular evaluation of future patients with arterial occlusion.

In our results, brain frailty was not independently associated with short-term outcomes in patients with ACI. Previous studies have shown that brain frailty is independently associated with the long-term outcomes in stroke patients, especially in patients with mild stroke [9]. There are two main reasons for this discrepancy: First, the patients we included were all AC-LAO, with larger infarct size and more severe symptoms. Early reperfusion is the key to good short-term recovery, so the effects of brain frailty are easily masked [36]. Second, this study did not analyze the effect of brain frailty on the long-term prognosis of patients with ACI. Brain frailty is an indicator of brain health and brain reserve, which may affect patients in later recovery [37]. In the future, we will continue to follow patients and investigate the relationship between brain frailty and long-term outcome of ACI in AC patients.

There are some limitations to this paper. First, this is a retrospective study with limited data content. Second, 10.6% of patients did not have baseline head CT or MRI and baseline brain frailty scores could not be obtained. Additionally, 25.4% of our patients only had baseline CT examinations, which limits the evaluation of brain frailty characteristics and interpretation of outcomes. Furthermore, long-term follow-up records of patient outcomes were not available to investigate the impact of brain frailty on outcomes in patients with AC-LAO.

This study is the first to include AC-LAO patients with brain frailty and assess the factors that influence brain frailty in AC-LAO patients, as well as the effect of brain frailty on stroke events. The results indicate that AC-LAO patients with old age, hypertension lasting > 5 years, and multiple vessel occlusion are significantly related to the occurrence of brain frailty. Furthermore, brain frailty is independently associated with the stroke events. Patients with AC-LAO combined with brain frailty should be treated more aggressively, and require more intensive follow-up. In future studies, we will further investigate the relationship between brain frailty and collateral circulation in patients with large artery stenosis/occlusion and the impact on the long-term patient prognosis.

### Electronic supplementary material

Below is the link to the electronic supplementary material.

Supplementary Material 1

## Data Availability

The datasets generated or analyzed during this study are available from the corresponding author on reasonable request.

## References

[1] Yassi N, Churilov L, Campbell BCV, Sharma G, Bammer R, Desmond PM (2015). The association between lesion location and functional outcome after ischemic stroke. Int J Stroke.

[2] Tziaka E, Christidi F, Tsiptsios D, Sousanidou A, Karatzetzou S, Tsiakiri A (2023). Leukoaraiosis as a predictor of Depression and Cognitive Impairment among Stroke survivors: a systematic review. Neurol Int.

[3] Delcourt C, Wang X, Zhou Z, Wardlaw JM, Mair G, Robinson TG (2020). Brain imaging abnormalities and outcome after acute ischaemic stroke: the ENCHANTED trial. J Neurol Neurosurg Psychiatry.

[4] Goyal M, Ospel JM, Kappelhof M, Ganesh A (2021). Challenges of Outcome Prediction for Acute Stroke Treatment decisions. Stroke.

[5] Gardener H, Caunca M, Dong C, Cheung YK, Alperin N, Rundek T (2018). Ideal Cardiovascular Health and biomarkers of subclinical brain aging: the Northern Manhattan Study. J Am Heart Assoc.

[6] Pase MP, Davis-Plourde K, Himali JJ, Satizabal CL, Aparicio H, Seshadri S (2018). Vascular risk at younger ages most strongly associates with current and future brain volume. Neurology.

[7] Gómez-Choco M, Mengual JJ, Rodríguez-Antigüedad J, Paré-Curell M, Purroy F, Palomeras E (2019). Pre-existing cerebral small vessel disease limits early recovery in patients with Acute Lacunar Infarct. J Stroke Cerebrovasc Dis.

[8] Hu X, De Silva TM, Chen J, Faraci FM (2017). Cerebral vascular Disease and Neurovascular Injury in ischemic stroke. Circ Res.

[9] Appleton JP, Woodhouse LJ, Adami A, Becker JL, Berge E, Cala LA (2020). Imaging markers of small vessel disease and brain frailty, and outcomes in acute stroke. Neurology.

[10] Bu N, Khlif MS, Lemmens R, Wouters A, Fiebach JB, Chamorro A (2021). Imaging markers of brain frailty and outcome in patients with Acute ischemic stroke. Stroke.

[11] Arba F, Inzitari D, Ali M, Warach SJ, Luby M, Lees KR (2017). Small vessel disease and clinical outcomes after IV rt-PA treatment. Acta Neurol Scand.

[12] Tschirret O, Moreno Legast G, Mansuy A, Mewton N, Buisson M, Hannoun S (2018). Impact of brain atrophy on early neurological deterioration and outcome in severe ischemic stroke treated by intravenous thrombolysis. Eur Neurol.

[13] Barnett K, Mercer SW, Norbury M, Watt G, Wyke S, Guthrie B (2012). Epidemiology of multimorbidity and implications for health care, research, and medical education: a cross-sectional study. Lancet.

[14] Flint AC, Bhandari SG, Cullen SP, Reddy AV, Hsu DP, Rao VA (2018). Detection of anterior circulation large artery occlusion in ischemic stroke using Noninvasive Cerebral Oximetry. Stroke.

[15] Kwon H-M, Lynn MJ, Turan TN, Derdeyn CP, Fiorella D, Lane BF (2016). Frequency, risk factors, and outcome of Coexistent Small Vessel Disease and Intracranial arterial stenosis: results from the stenting and Aggressive Medical Management for preventing recurrent stroke in intracranial stenosis (SAMMPRIS) trial. JAMA Neurol.

[16] Song J, Kim K-H, Jeon P, Kim Y-W, Kim D-I, Park Y-J (2021). White matter hyperintensity determines ischemic stroke severity in symptomatic carotid artery stenosis. Neurol Sci.

[17] Zerna C, Yu AYX, Modi J, Patel SK, Coulter JI, Smith EE (2018). Association of White Matter Hyperintensities with short-term outcomes in patients with minor cerebrovascular events. Stroke.

[18] de Havenon A, Wong K-H, Elkhetali A, McNally JS, Majersik JJ, Rost NS (2019). Carotid artery stiffness accurately predicts White Matter Hyperintensity 20 years later: a secondary analysis of the atherosclerosis risk in the Community Study. AJNR Am J Neuroradiol.

[19] Zhang K, Li T, Tian J, Li P, Fu B, Yang X (2020). Subtypes of anterior circulation large artery occlusions with acute brain ischemic stroke. Sci Rep.

[20] Cui J, Yang J, Zhang K, Xu G, Zhao R, Li X (2021). Machine learning-based Model for Predicting incidence and severity of Acute ischemic stroke in anterior circulation large vessel occlusion. Front Neurol.

[21] Farrell C, Chappell F, Armitage PA, Keston P, Maclullich A, Shenkin S (2009). Development and initial testing of normal reference MR images for the brain at ages 65–70 and 75–80 years. Eur Radiol.

[22] IST-3 collaborative group (2015). Association between brain imaging signs, early and late outcomes, and response to intravenous alteplase after acute ischaemic stroke in the third International Stroke Trial (IST-3): secondary analysis of a randomised controlled trial. Lancet Neurol.

[23] Fazekas F, Chawluk JB, Alavi A, Hurtig HI, Zimmerman RA (1987). MR signal abnormalities at 1.5 T in Alzheimer’s dementia and normal aging. AJR Am J Roentgenol.

[24] Pantoni L (2010). Cerebral small vessel disease: from pathogenesis and clinical characteristics to therapeutic challenges. Lancet Neurol.

[25] Scharf EL, Graff-Radford J, Przybelski SA, Lesnick TG, Mielke MM, Knopman DS (2019). Cardiometabolic Health and Longitudinal Progression of White Matter Hyperintensity: the Mayo Clinic Study of Aging. Stroke.

[26] Tariq S, Barber PA (2018). Dementia risk and prevention by targeting modifiable vascular risk factors. J Neurochem.

[27] Pathak GA, Barber RC, Phillips NR (2021). Multiomics Investigation of Hypertension and White Matter Hyperintensity as a source of vascular dementia or a comorbidity to Alzheimer’s Disease. Curr Alzheimer Res.

[28] Shi Y, Guo L, Chen Y, Xie Q, Yan Z, Liu Y (2021). Risk factors for ischemic stroke: differences between cerebral small vessel and large artery atherosclerosis aetiologies. Folia Neuropathol.

[29] Yoshida J, Yamashita F, Sasaki M, Yoshioka K, Fujiwara S, Kobayashi M (2020). Adverse effects of pre-existing cerebral small vessel disease on cognitive improvement after carotid endarterectomy. Int J Stroke.

[30] Wang X-Y, Lyu J-H, Zhang S-H, Duan C-H, Duan Q, Ma X-X (2022). Severity of Intracranial large artery Disease correlates with cerebral small Vessel Disease. J Magn Reson Imaging.

[31] Shu M-J, Zhai F-F, Zhang D-D, Han F, Zhou L, Ni J (2021). Metabolic syndrome, intracranial arterial stenosis and cerebral small vessel disease in community-dwelling populations. Stroke Vasc Neurol.

[32] Liu M, Nie Z-Y, Li R-R, Zhang W, Wang H, He Y-S (2018). Correlation of Brain Perfusion with White Matter Hyperintensity, Brain Atrophy, and Cognition in patients with posterior cerebral artery stenosis and subjective cognitive decline. Med Sci Monit.

[33] Fischer U, Hsieh-Meister K, Kellner-Weldon F, Galimanis A, Yan X, Kaesmacher J (2020). Symptomatic and asymptomatic intracranial atherosclerotic stenosis: 3 years’ prospective study. J Neurol.

[34] Hurford R, Wolters FJ, Li L, Lau KK, Küker W, Rothwell PM (2020). Prevalence, predictors, and prognosis of symptomatic intracranial stenosis in patients with transient ischaemic attack or minor stroke: a population-based cohort study. Lancet Neurol.

[35] Zhou J-Y, Shi Y-B, Xia C, Lu C-Q, Tang T-Y, Lu T (2022). Beyond collaterals: brain frailty additionally improves prediction of clinical outcome in acute ischemic stroke. Eur Radiol.

[36] Campbell BCV, Mitchell PJ, Churilov L, Yassi N, Kleinig TJ, Dowling RJ (2020). Effect of intravenous tenecteplase dose on cerebral reperfusion before thrombectomy in patients with large vessel occlusion ischemic stroke: the EXTEND-IA TNK Part 2 Randomized Clinical Trial. JAMA.

[37] Duan Q, Lyu J, Cheng K, Wang X, Meng Z, Wu X (2023). MRI Assessment of Brain Frailty and clinical outcome in patients with acute posterior perforating artery infarction. J Magn Reson Imaging.

