# Supplementary material for: Brain frailty associated with stroke events in anterior circulation large artery occlusion
Source: BMC Neurol. 2024 Mar 18;24:97. doi: 10.1186/s12883-024-03566-7 (PMC10946158; doi:10.1186/s12883-024-03566-7)
Supplement: Supplementary file 1 — Supplementary Material 1 [file 12883_2024_3566_MOESM1_ESM.docx]

Table S1 Demographic and clinical characteristics of study population according to imaging markers of brain frailty

| Variables | Total N=983 | WMH | | Cerebral atrophy | | Old infarction lesions | |
| --- | --- | --- | --- | --- | --- | --- | --- |
|  |  | N=269 | *P* Value | N=398 | *P* Value | N=671 | *P* Value |
| Age, y, median (IQR) | 62 (14) | 66 (12) | <0.001 | 66 (11) | <0.001 | 64 (14) | <0.001 |
| Age categories | |  | <0.001 |  | <0.001 |  | <0.001 |
| ≤55 | 316 (32.1) | 37 (13.8) |  | 62 (15.6) |  | 177 (26.4) |  |
| 56∼65 | 334 (34.0) | 87 (32.3) |  | 130 (32.7) |  | 237 (35.3) |  |
| >66 | 333 (33.9) | 145 (53.9) |  | 206 (51.8) |  | 257 (38.3) |  |
| Male, n (%) | 667 (67.9) | 171 (63.6) | 0.077 | 291 (73.1) | 0.004 | 457 (68.1) | 0.803 |
| Smoking, current, n (%) | 344 (35.0) | 77 (28.7) | 0.012 | 129 (32.6) | 0.190 | 214 (32.0) | 0.004 |
| Drinking, current, n (%) | 279 (28.4) | 68 (25.4) | 0.202 | 111 (28.0) | 0.847 | 176 (26.3) | 0.04 |
| Hypertension, n (%) | 643 (65.4) | 201 (74.7) | <0.001 | 272 (68.3) | 0.111 | 462 (68.9) | 0.001 |
| Duration of hypertension >5y, n (%) | 418 (42.5) | 154 (57.2) | <0.001 | 202 (50.8) | <0.001 | 315 (46.9) | <0.001 |
| Diabetes mellitus, n (%) | 251 (25.5) | 75 (27.9) | 0.300 | 109 (27.4) | 0.272 | 193 (28.8) | 0.001 |
| Diabetes mellitus time>5y, n (%) | 110 (11.2) | 34 (12.6) | 0.376 | 50 (12.6) | 0.260 | 86 (12.8) | 0.018 |
| coronary heart disease, n (%) | 150 (15.3) | 49 (18.2) | 0.114 | 82 (20.6) | <0.001 | 112 (16.7) | 0.067 |
| Atrial fibrillation, n (%) | 55 (5.6) | 18 (6.7) | 0.359 | 33 (8.3) | 0.002 | 47 (7.0) | 0.005 |
| Prior TIA, n (%) | 67 (6.8) | 17 (6.3) | 0.705 | 29 (7.3) | 0.629 | 42 (6.3) | 0.310 |
| Prior stroke, n (%) | 365 (37.1) | 133 (49.4) | <0.001 | 205 (51.5) | <0.001 | 358 (53.4) | <0.001 |
| Hyperlipidemia, n (%) | 315 (32.0) | 77 (28.6) | 0.158 | 116 (29.1) | 0.108 | 202 (30.1) | 0.056 |
| LDL-C, mmol/L, median (IQR) | 2.40 (1.15) | 2.37 (1.01) | 0.307 | 2.26 (1.07) | 0.003 | 2.32 (1.08) | 0.003 |
| ApoA1, g/L, median (IQR) | 1.13 (0.27) | 1.13 (0.27) | 0.026 | 1.13 (0.27) | 0.490 | 1.13 (0.27) | 0.370 |
| ApoB, g/L, median (IQR) | 0.95 (0.31) | 0.95 (0.32) | 0.635 | 0.94 (0.33) | 0.006 | 0.95 (0.33) | 0.001 |
| HCY, umol/L, median (IQR) | 16.0 (9.7) | 16.00 (8.40) | 0.924 | 16.10 (9.80) | 0.370 | 16.0 (9.3) | 0.389 |
| occluded vessels position | |  | 0.263 |  | 0.031 |  | 0.034 |
| MCA occlusion | 523 (53.2) | 141 (52.4) |  | 195 (49.0) |  | 350 (52.2) |  |
| ICA occlusion | 289 (29.4) | 73 (27.1) |  | 120 (30.2) |  | 190 (28.3) |  |
| MCA+ICA occlusion | 171 (17.4) | 55 (20.4) |  | 83 (20.9) |  | 131 (19.5) |  |
| multiple vessel occlusion, n (%) | 246 (25.0) | 79 (29.4) | 0.054 | 118 (29.6) | 0.006 | 190 (28.3) | <0.001 |
| ACA occlusion, n (%) | 209 (21.3) | 60 (22.3) | 0.624 | 91 (22.9) | 0.311 | 153 (22.8) | 0.083 |
| PC occlusion, n (%) | 198 (20.1) | 59 (22.0) | 0.376 | 95 (23.9) | 0.015 | 155 (23.1) | 0.001 |
| Stroke events, median (IQR) | 1 (1) | 1 (1) | <0.001 | 1 (1) | 0.009 | 1 (1) | <0.001 |
| ACI, n (%) | 645 (65.6) | 190 (70.6) | 0.042 | 227 (57.0) | <0.001 | 426 (63.5) | 0.039 |
| Frontal lobe infarction, n (%) | 362 (36.8) | 102 (37.9) | 0.663 | 128 (32.2) | 0.012 | 230 (34.3) | 0.015 |
| temporal lobe infarction, n (%) | 299 (30.4) | 85 (31.6) | 0.621 | 95 (23.9) | <0.001 | 190 (28.3) | 0.036 |
| parietal infarction, n (%) | 349 (35.5) | 104 (38.7) | 0.204 | 121 (30.4) | 0.006 | 217 (32.3) | 0.002 |
| basal ganglia infarction, n (%) | 320 (32.6) | 81 (30.1) | 0.316 | 95 (23.9) | <0.001 | 193 (28.8) | <0.001 |
| Posterior circulation infarction, n (%) | 151 (15.4) | 51 (19.0) | 0.056 | 52 (13.1) | 0.097 | 95 (14.2) | 0.120 |
| The shape of the lesion in the occluded vessel | |  | 0.011 |  | 0.105 |  | 0.004 |
| No lesions or scattered | 190 (19.3) | 39 (14.5) |  | 75 (18.8) |  | 117 (17.4) |  |
| Patchy | 603 (61.3) | 185 (68.8) |  | 258 (64.8) |  | 435 (64.8) |  |
| Large flakes | 190 (19.3) | 45 (16.7) |  | 65 (16.3) |  | 119 (17.7) |  |
| Complication, n (%) | 167 (17.0) | 56 (20.8) | 0.050 | 81 (20.4) | 0.021 | 121 (18.0) | 0.201 |
| pneumonia, n (%) | 108 (11) | 38 (14.1) | 0.053 | 60 (15.1) | 0.001 | 79 (11.8) | 0.247 |
| hypoalbuminemia, n (%) | 28 (2.8) | 10 (3.7) | 0.315 | 18 (4.5) | 0.009 | 22 (3.3) | 0.234 |
| NIHSS score on admission, median (IQR) | 4 (7) | 5 (8) | 0.010 | 4 (7) | 0.735 | 4 (7) | 0.924 |
| NIHSS score at discharge, median (IQR) | 3 (8) | 4 (8) | 0.001 | 3 (8) | 0.734 | 3 (8) | 0.823 |
| mRS score on admission, median (IQR) | 2 (3) | 3 (3) | 0.010 | 2 (3) | 0.784 | 2 (3) | 0.735 |
| mRS score at discharge, median (IQR) | 2 (3) | 3 (3) | 0.002 | 2 (3) | 0.554 | 2 (3) | 0.528 |

Abbreviations: WMH = white matter hyperintensity; IQR = interquartile range; LDL-C = Low density lipoprotein cholesterol; ApoA1 = apolipoprotein A1; ApoB, = apolipoprotein B; HCY = homocysteine; MCA = middle cerebral artery; ICA = internal carotid artery; ACA = Anterior cerebral artery; PC = Posterior circulation; ACI = acute cerebral infarction.

Table S2. Binary logistic regression analysis of WMH

|  | n (%) | WMH n (%) | logistic regression | |
| --- | --- | --- | --- | --- |
|  |  |  | OR (95%CI) | *p* value |
| n | 983 | 269 (27.4) |  |  |
| Age, years |  |  |  | <0.001 |
| ≤55 | 316 (32.1) | 37 (11.7) | 1 |  |
| 56-65 | 334 (34.0) | 87 (26.0) | 2.49 (1.60, 3.86) | <0.001 |
| ≥66 | 333 (33.9) | 145 (43.5) | 5.39 (3.48, 8.32) | <0.001 |
| Hypertension time (>5y) | 418 (42.5) | 154 (36.8) | 1.69 (1.12, 2.58) | 0.014 |
| ApoA1, median (IQR) | 1.13 (0.27) | 1.13 (0.27) | 1.94 (1.003, 3.75) | 0.049 |
| The shape of the lesion in the occluded vessel | | | | 0.008 |
| No lesions or scattered | 190 (19.3) | 39 (20.5) | 1 |  |
| Patchy | 603 (61.3) | 185 (30.7) | 1.60 (1.03, 2.48) | 0.035 |
| Large flakes | 190 (19.3) | 45 (23.7) | 0.92 (0.51, 1.63) |  |

Abbreviations: WMH = white matter hyperintensity; OR = odds ratio; CI = confidence interval; ApoA1 = apolipoprotein A1; IQR = interquartile range.

Table S3. Binary logistic regression analysis of cerebral atrophy

|  | n (%) | cerebral atrophy, n (%) | logistic regression | |
| --- | --- | --- | --- | --- |
|  |  |  | OR (95%CI) | *p* value |
| n | 983 | 398 (40.5) |  |  |
| Age, years |  |  |  | <0.001 |
| ≤55 | 316 (32.1) | 62 (19.6) | 1 |  |
| 56-65 | 334 (34.0) | 130 (38.9) | 2.52 (1.74, 3.64) | <0.001 |
| ≥66 | 333 (33.9) | 206 (61.9) | 6.63 (4.54, 9.69) | <0.001 |
| Gender (male) | 667 (67.9) | 291 (43.6) | 2.00 (1.47, 2.74) | <0.001 |
| Multiple vessel occlusion | 246 (25.0) | 118 (48.0) | 1.48 (1.07, 2.05) | 0.018 |
| Hypertension time (>5y) | 418 (42.5) | 202 (48.3) | 1.39 (1.04, 1.86) | 0.027 |
| Basal ganglia infarction | 320 (32.6) | 95 (29.7) | 0.50 (0.36, 0.70) | <0.001 |
| Temporal lobe infarction | 299 (30.4) | 95 (31.8) | 0.67 (0.47, 0.94) | 0.019 |
| Pneumonia | 108 (11.0) | 60 (55.6) | 1.75 (1.1, 2.76) | 0.017 |
| Hypoalbuminemia | 28 (2.8) | 18 (64.3) | 2.48 (1.02, 6.00) | 0.045 |

Abbreviations: OR=odds ratio; CI=confidence interval.

Table S4: Binary logistic regression analysis of old infarction lesions

|  | n (%) | Old lesions, n(%) | logistic regression | |
| --- | --- | --- | --- | --- |
|  |  |  | OR(95%CI) | *p* value |
| n | 983 | 365 (37.1) |  |  |
| Age, years |  |  |  | <0.001 |
| ≤55 | 316 (32.1) | 177 (56.0) | 1 |  |
| 56-65 | 334 (34.0) | 237 (71.0) | 1.61 (1.14, 2.28) | 0.007 |
| ≥66 | 333 (33.9) | 257 (77.2) | 2.20(1.52, 3.18) | <0.001 |
| Diabetes mellitus | 251 (25.5) | 193 (76.9) | 1.57 (1.10, 2.23) | 0.013 |
| PC occlusion | 198 (20.2) | 155 (78.3) | 1.70 (1.15, 2.52) | 0.008 |
| Multiple vessel occlusion | 246 (25.0) | 190 (77.2) | 1.68 (1.17, 2.40) | 0.005 |
| Hypertension time (>5y) | 523 (53.2) | 199 (38.0) | 1.46 (1.07, 1.98) | 0.016 |
| Basal ganglia infarction | 320 (32.6) | 193 (60.3) | 0.64 (0.46, 0.89) | 0.008 |
| The shape of the lesion in the occluded vessel | | | | <0.001 |
| None or scattered | 190 (19.3) | 117 (61.6) | 1 |  |
| Patchy | 603 (61.3) | 435 (72.1) | 2.20 (1.50, 3.23) | <0.001 |
| Large flakes | 190 (19.3) | 119 (62.6) | 1.79 (1.07, 3.00) | 0.026 |

Abbreviations: OR=odds ratio; CI=confidence interval; PC=Posterior circulation.

Table S5: Multiple linear regression of variables and mRS score at discharge

|  | n (%) | multiple linear regression | |
| --- | --- | --- | --- |
|  |  | MD (95%CI) 𝛃 | *p* value |
| n, median (IQR) | 645, 3(3) |  |  |
| Past TIA | 41 (6.4) | -0.62 (-0.96, -0.29) | <0.001 |
| NIHSS score at admission, median (IQR)^a^ | 6 (8) | 0.53 | <0.001 |
| complication | 124 (19.2) | 0.35 (0.13, 0.57) | 0.002 |
| Basal ganglia infarction | 320 (49.6) | 0.24 (0.07, 0.40) | 0.006 |
| Occluded vessel position, median (IQR)^a^ | 1 (1) | 0.11 | 0.024 |
| Occluded Vessel Count, median (IQR)^a^ | 1 (1) | -0.13 | 0.007 |
| The shape of the lesion in the occluded vessel (Scattered, patchy, large flakes) | 61 (9.5),  414 (64.2),  170 (26.4) | 0.22 (0.106, 0.38) | 0.008 |

Occluded vessel position was divided into 8 categories, 1= Unilateral MCA occlusion, 333 (51.6); 2=Unilateral ICA occlusion, 153 (23.7); 3=Bilateral MCA occlusion, 34 (5.3); 4=Bilateral ICA occlusion, 6 (0.9); 5= Bilateral MCA+ Unilateral ICA occlusion, 17 (2.6); 6= Bilateral ICA+ Unilateral MCA occlusion, 0; 7=Unilateral MCA +Unilateral ICA occlusion, 81 (12.6); 8= Unilateral ICA + Bilateral MCA occlusion, 21 (3.3).

Abbreviations: MD = mean difference; IQR = interquartile range; TIA = transient ischemic attack; MCA = middle cerebral artery; ICA = internal carotid artery.
